# Formulating tobacco control policies: How can local governments contribute?

**DOI:** 10.18332/tpc/191844

**Published:** 2024-10-17

**Authors:** Sophie J. A. Jooren, Jeroen Bommelé, Eefje Willemse, Maria W. J. Jansen, Marc C. Willemsen

**Affiliations:** 1 Department of Health Promotion, CAPHRI Care and Public Health Research Institute Maastricht University, Maastricht, the Netherlands; 2 The Netherlands Expertise Centre for Tobacco Control, Trimbos Institute, Netherlands Institute of Mental Health and Addiction, Utrecht, the Netherlands; 3 Department of Health Services Research, CAPHRI Care and Public Health Research Institute, Maastricht University, Maastricht, the Netherlands

**Keywords:** municipalities, local tobacco control, content analysis

## Abstract

**INTRODUCTION:**

Due to a continuing international trend of decentralization of public health policies, local governments are given an increasingly important role in tobacco control. The process of developing local-level tobacco control policies is an underexplored topic. This study uses grant applications as a data source to gain insight into the planning, development and proposed implementation of local tobacco control policies by regional public health departments in the Netherlands.

**METHODS:**

Grant applications of 24 regional public health departments were analyzed using the second stage of the rational policy cycle, a four-stages policy model about the decisions made by local policy makers during the policy process. We coded the applications with open and axial coding.

**RESULTS:**

Public health departments formulated four main goals for tobacco control: adding tobacco control policies to existing local policy documents, creating smoke-free (child) environments, developing and improving access to smoking cessation care, and participating in media campaigns. Public health departments often specify tobacco control aims and involve partners in reaching these aims. However, the grant applications lacked information about implementing these tobacco control policies.

**CONCLUSIONS:**

The information on implementation strategies and process evaluation, as well as the (evidence-based) legitimation for the policy choices, needs improvement. Under the current conditions, which include the brief explanation the departments received, an unclear mandate, insufficient funding, and local restricting factors such as time and knowledge, significant contributions to tobacco control policy cannot be expected from local governments.

## INTRODUCTION

Tobacco use is one of the world’s largest preventable causes of death^1^. To address this, the WHO introduced the WHO Framework Convention on Tobacco Control Treaty (WHO FCTC) in 2003. Its objective is to protect present and future generations from the health-related, social, environmental, and economic consequences associated with tobacco use and tobacco smoke exposure. Both national and local governments have an important role in commitment and contributing to the WHO FCTC^2^.

Due to a continuing international trend of decentralization of public (health) policies in Europe, local governments at the municipality level are given more responsibilities regarding tobacco control^3-5^. For instance, Denmark has shifted significant responsibility for prevention and health promotion to the municipalities^6^. Danish municipalities are now accountable for smoking cessation initiatives, with support from the national government^6^. However, only a relatively small body of literature discusses the development and implementation of local tobacco control policies^7-9^.

The present study has three objectives: 1) to investigate what local tobacco control policy choices local public health departments make regarding tobacco control policy formulation, 2) how these choices are substantiated, and 3) how these choices and substantiations can be explained.

To gain insight into these aims, we focus on the Netherlands where tobacco control policies are relatively well developed^2,10^. Plain cigarette packaging has been introduced; there is a stepped duty increase on packs of cigarettes and rolling tobacco; a national smoking ban applies in all indoor public buildings and the hospitality industry; and outdoor school areas are smoke-free by law^11,12^. The law does not mandate other outdoor areas to be smoke-free. The Netherlands is one of the few countries worldwide to have implemented the WHO FCTC’s tobacco control measures to the fullest extent^2^, making it an interesting case study to gain insight into the potential of local tobacco control policies in the context of national tobacco control policy.

### Tobacco control in the Netherlands

In 2019, the government of the Netherlands introduced the National Prevention Agreement. The National Prevention Agreement describes long-term public health goals in the fields of tobacco control, reduction of alcohol use, and prevention of obesity^13^. The policy target for smoking is to achieve a smoke-free society by 2040, meaning that 5% of adults and 0% of children smoke. Currently, smoking prevalence is 18.9% among adults (2022) and 9.5% among children (2021)^14,15^. Although most of the tobacco control measures are aimed at the government and the national tobacco discouragement policy, the national prevention agreement acknowledges that local governments also play a role in reducing smoking.

Municipalities in the Netherlands are responsible for public health policies, health protection, health promotion, and health monitoring. These responsibilities are laid down in the Dutch Public Health Act^16,17^. Municipalities are expected to contribute to the aim of the National Prevention Agreement by implementing local tobacco control policies, particularly through establishing smoke-free outdoor environments, organizing campaigns about the dangers of tobacco, and supporting effective and accessible smoking cessation care^13,18^. The municipalities receive support from regional public health departments (in Dutch: GGD’en). Regional public health departments are joint arrangements and are governed by the aldermen of the participating municipalities. Currently, there are 25 regional public health departments covering the whole country^19^. A typical public health department is responsible for 6–26 municipalities, including a total population of approximately 0.6–0.8 million.

A few studies have been conducted about implementing local tobacco control policies in the Netherlands by contextualizing them within the wider scope of their responsibilities for public health. Huijsman et al.^20^ found that policymakers in municipalities failed to prioritize local tobacco control policies over other health risk behaviors such as being overweight, alcohol consumption, and drug use. Mulder et al.^18^ observed that local tobacco control policy intentions tend to be ‘hidden’ within broader municipality health policies. Most of the time money that municipalities can spend on substance abuse policy go to alcohol and drugs^18^. Recently, Bruijn and Hessels^21^ studied a representative geographical sample of 49 Dutch municipalities and found that only 53% of municipalities had set tobacco control policy targets (e.g. the number of smoke-free areas created). Only a third of these had translated those targets into concrete actions and measurable results^21^. Few municipalities have given tobacco control an explicit place in their local health policy. In addition, only a quarter of the municipalities allocated a budget for tobacco control policies in the local health policy plans^21^.

In summary, although the national government expects municipalities to contribute to tobacco control, previous research showed that municipalities seem reluctant to do so^18,20,21^. Several federal countries, including the United States (US), Canada, and Australia, operate under a governmental system in which municipalities have significant legislative power, enabling them to enact tobacco control policies independently from national governments. However, such a mandated approach appears to be less prevalent in Europe. In the Netherlands, the national government has limited legislative power over local governments, similar to many other countries in Europe. Generally, there are two ways for the Dutch national government to influence local tobacco control policymaking: by putting moral pressure on local governments to take responsibility to allocate more of their budget to tobacco control, or by collaborating with public health departments and providing them with dedicated tobacco control budgets or grants which they can use to activate municipalities. This study examines this second strategy by examining the formulation and adoption of tobacco control policies.

### Rational policy model

Various policy models can be used to study policy-making within public health, such as tobacco control. In this article, we use the rational model of policymaking to structure the content of public health departments’ grant applications. The stages of this model are: 1) identifying a socially relevant problem, 2) policy formulation, 3) policy implementation, and 4) policy evaluation^22^. The ‘rational policy cycle’ assumes that policymakers go through ‘logical’ stages to make appropriate decisions^22,23^. The sequence of stages is depicted in a somewhat simplified manner and as a one-way process. However, in reality, policymaking is often an iterative learning process from one stage to the next and back again, involving a multitude of stakeholders.

In the local context, stage 2 entails developing and adopting a policy on a socially relevant problem with political agreement by the municipal council. Stage 2 is evidence-informed, meaning that the policy plan is based on available evidence in the light of feasibility, practicability, affordability, and acceptability. In other words, which approach works best according to scientific knowledge (best-proven evidence), and is the municipality willing and able to implement, pay for, and legitimize this? This crucial stage is understudied within the scientific literature and might help to understand the variation across countries in the formulation and adoption of tobacco control policies^24^.

## METHODS

### Context, sampling strategy, and data collection

We assessed the content of the public health department’s tobacco control policy plans that were eligible for local tobacco control funding provided by the national. In 2019, the Ministry of Health, Welfare, and Sport organized a grant application process through the umbrella association of the regional public health departments (In Dutch: GGD GHOR Nederland). Public health departments were given the opportunity to apply for this grant (80000 euros for a two-year period). The following topics qualified for the grant: to prioritize smoke-free policy integrated into public health regulatory documents and prevention agreements, to create smoke-free environments, to focus on low socioeconomic status groups, and to stimulate effective and accessible smoking cessation care^25^. In 2021, twenty-four public health departments had applied. The 24 applications received from GGD GHOR Nederland covered almost all municipalities in the Netherlands (343 of the 355 municipalities) and were all included in our study.

All applications used the same mandatory format: 1) general information about the municipalities in the public health department, 2) current municipal activities concerning a Smoke-Free Generation, 3) a summary of the plan, 4) cooperation being undertaken with other public health sectors, 5) incorporating tobacco control into the municipalities’ regular work activities, 6) incorporating the plans within existing policies on reducing problematic alcohol consumption and being overweight, and 7) any tools and instruments made by the public health department of municipalities for this topic during the course of the grant. The grant applications varied between 3 and 10 pages in length, included between 1200 and 3500 words, and were written in Dutch.

### Qualitative approach and data analysis

We operationalized stage 2 of the rational policy cycle, ‘formulation of a policy plan’, by analyzing the grant applications. Stage 2 consists of several steps: assignment analysis (who should be involved?), problem and causal analysis (description of the problem and the interpretation of the causal and unproven-causal relationships), goals/objectives, selection of policy instruments (laws and regulations, price policy or incentives, and communication), implementation design (the roadmap to show what, where, when, how, and by whom activities have to be implemented), and costs and benefits (whether the policy is effective or cost-effective)^22^.

The first (SJ) and third author (EW) performed the analysis according to a combination of open and axial coding principles^26^. Coding was conducted independently using MAXQDA (20.4.2.). First, during open coding, the researchers investigated the data and contributed relevant codes to the data. For instance, the role of public health departments was divided into several open codes, such as policy implementation, policy advising, and coordinating functions. Second, during axial coding, the researchers structured the codes into more overarching themes. For example, all the different roles of the public health departments were put into the overarching theme ‘role public health departments’. The researchers engaged in discussions regarding the differences in their codes. Finally, during the thematic analysis, the researchers identified the most important themes for the topic studied. Collaboration (between themes and with other partners), justification for the topic, goals on the local level, different policy documents, smoke-free environments, smoking cessation care, campaigns, and evaluation were major topics. As a final step, these themes were then classified according to the six steps of stage 2 of the rational policy cycle. The suitability and categorization of the codes and themes were continuously deliberated on by all the authors, making adjustments if necessary.

### Ethics

Regional public health departments were approached by GGD GHOR Nederland for the use of their grant applications and permission was obtained. Ethical approval was received from the Ethics Review Committee of the Faculty of Health, Medicine, and Life Sciences of Maastricht University (FHML-REC/2022/021).

## RESULTS

We present the relevant findings according to the different steps of the second stage of the rational policy cycle.

### Step 1: Assignment analysis

Six out of 24 public health departments intended to collaborate with other health departments to work on local tobacco control policies. Fourteen public health departments wrote about working together with several, not yet specified but optional, other departments within the municipality, for example: ‘Within municipalities, we are looking for cooperation with various departments. In addition to public health, we want to cooperate with, for example, social domain, participation and work, area teams, and youth, education, and sport’.

### Step 2: Problem and causal analysis

Only four out of 24 applications included local smoking rates. Among these four, prevalence rates were reported by education level (n=3), by sex/gender (n=3), among youth (n=2), among people with a migration background (n=1), or overall trends in smoking among adults and youths (n=1). Ten grant applications included data on the number of smoke-free environments that were already in place within their municipalities. Some applications included perceived causal relationships. Four public health departments associated smoking with underlying factors such as stress and poverty. Two emphasized the need for a healthy environment to promote overall well-being. Two highlighted the impact of ‘seeing smoking’ on smoking behavior, while one discussed the consequences for children when their mothers smoke.

### Step 3: Goals/Objectives

We identified five different goals in the grant applications: 1) contributing to reaching the national target of a Smoke-Free Generation in 2040 (n=24), 2) including smoking in local policy documents (n=24), 3) obtaining more smoke-free environments (n=24), 4) realizing accessible smoking cessation care (n=17), and 5) implementing media campaigns (n=22). Within the third goal, i.e. smoke-free environments, half of the public health departments merely discussed smoke-free child environments as part of a broad policy package, while the other half formulated specific goals for smoke-free areas, such as ‘The region has created 25 new smoke-free environments at the end of the grant period’.

Overall, we found that tobacco control in general, and more specifically a Smoke-Free Generation, smoke-free environments, and smoke-free child environments, were targets for policy formulation. Some regions focus on tobacco control policies in general, e.g. ‘After two years, at least ten municipalities will have tobacco discouragement in the new local health policy nota’. Others specifically targeted one part of tobacco control policies, such as smoke-free environments, ‘We ask municipalities whether they want to include making (child) environments smoke-free in their local regulation or environmental vision’.

### Step 4: Selection of policy instruments

In order to realize smoke-free environments, laws, regulations, incentives, and communication were the preferred policy instruments. Some proposed to include smoke-free environments in general local bylaws that pertain to municipal regulations related to public order and safety (n=11) or wanted to incorporate smoke-free environments into subsidy conditions (n=9), e.g. sports associations. Communication strategies were proposed as a policy instrument for both campaigns and smoke-free environments. All public health departments expressed their intention to communicate the concept of smoke-free environments by installing nationally designed signs in outdoor areas, providing information about the Smoke-Free Generation. Communication appears to be the favored policy instrument for smoking cessation care.

### Step 5: Implementation design

Public health departments and municipalities plan to integrate tobacco control in broader, more general policies that already exist at the local level, such as a general municipal health policy (n=19), spatial planning and environmental visions plans (n=13), regional or local public health prevention programs or agreements (n=12), and municipal grant conditions for local organizations (n=10). More outdoor smoke-free environments were associated with locations where children are present, including sports associations (n=23), schools (n=21), play and recreation areas (n=19), childcare facilities (n=13), petting zoos (n=10), and to a less extent inside environments including community homes, care institutions, and businesses. Furthermore, they propose to improve smoking cessation care by mapping the available cessation support in the region with a guide (n=12), making a care pathway that describes what healthcare workers can do, such as referring smokers to specialized cessation counseling (n=4) or providing information on the website of the public health department or municipality (n=2). Public health departments specified the further implementation by participating in national campaigns. Six public health departments provided more detailed information, mainly about their own regional campaigns.

Most applications provided very little information about the proposed process for implementing local tobacco control. All public health departments provided general information about the involved municipalities, the activities done so far in the region regarding smoking, and the proposed activities for the upcoming period of the grant application for two years. The described activities were specified in the context of a specific location, such as the school environment. How the activities have to be implemented was hardly mentioned. Public health departments often mention the goal and the involved partner but do not know how to go about reaching that goal specifically. For example, public health departments mentioned that they want to implement smoke-free places but not how this should be implemented. They also did not mention anything about legal aspects, such as (non)compliance.

Regarding the partners involved, we observed many differences between the regions. Generally, the responsibility for achieving the goals outlined in the grant applications was shared between public health departments and municipalities. Municipalities were required to cooperate with local partners, including sports associations, schools, hospitals, playgrounds, health insurers, and health professionals. Additionally, 22 public health departments proposed to align with existing general health-promoting programs that were already in place within the municipality. In most cases (n=19), public health departments proposed to take on the role of policy advisors, delegating executive tasks to the municipality or local partners. However, we identified 11 public health departments that expressed a desire for a more executive role, such as ‘Inspiring (healthcare) professionals in different neighborhoods to support the Smoke-Free Generation’ or ‘Establishing a regional network of partners’. In addition to these roles, some public health departments said they wanted to take on motivating roles, including policy supporter (n=9), connector (n=13), stimulator (n=11), motivator (n=9), agenda setter (n=8), and project leader (n=3). Other roles we identified were coordinator (n=7) and monitor (n=9).

### Step 6: Progress evaluation

All public health departments had to write a mandatory semi-annual progress report for this grant. Only nine public health departments addressed additional forms of monitoring. Generally, plans only included broad statements such as: ‘Monitoring with the results of the national health monitor’ or ‘The project leaders are responsible for monitoring the results and connect with existing monitors of colleagues, national parties, and cooperation partners’. Two public health departments explained their monitoring plans more elaborately. One public health department described that the region will monitor the municipal starting point and end result to assess their progress. One public health department had planned to make an appealing, innovative website for visible monitoring of smoke-free places within the region.

## DISCUSSION

The aim of this study was to explore, using the second stage of the rational policy cycle, what choices local public health departments make regarding tobacco control policy, how these choices are substantiated, and how these substantiations can be explained.

Public health departments prioritized the creation of more smoke-free environments, implementation of educational campaigns, and enhancing smoking cessation care by improving access to smoking cessation services. The choices made by public health departments broadly align with the recommendations in the National Prevention Agreement.

The emphasis on smoke-free areas for children can be attributed to national regulations that prioritize smoke-free environments for children. School grounds, for example, were made smoke-free through national regulations in 2020^27^. The focus of public health departments thus began with helping schools in the implementation and enforcement of the national ban before extending activities to venues other than school campuses. The overall tendency to focus on youth-related settings is evident across Europe, as demonstrated by Mlinarić et al.^28^ in a study comparing seven European cities. Additionally, an overview from the European Commission regarding smoke-free legislation and its implementation in Europe shows this^29^. Arguments such as the child protection framework can ensure that policymakers are less influenced by the economic interests of smoking because protecting children is more important^30^.

Since the grant could not be used to create new locally/regionally oriented campaigns, only six public health departments prioritized investing in existing local campaigns.

With regard to smoking cessation care, the national policy developments of decentralization play an important role, given that smoking cessation support is predominantly left to the free market, where health insurance providers play a pivotal role^13,31^. Neither the national nor the local government indicated a role for local governments to negotiate with health insurances concerning smoking cessation care.

### National policy context and local factors

While the public health departments did elaborate on how the choices for policies were made, they did not adequately explain and scientifically underpin why they made these choices. Furthermore, information about implementation and evaluation was lacking. This finding was also noted by Bruijn and Hessels^21^ who found that only a third of the 51 Dutch municipalities in their study have given tobacco control an explicit place in their local health policy. Part of why there was so little explanation of the choices that the public health departments made, has to do with the specific context in which the plans had to be written. Especially: 1) the brief explanation the departments received and the unclear mandate they were given, 2) the small amount of funding, and 3) local factors such as time and knowledge. First, it should be noted that, in the current Dutch policy context, the public health departments and municipalities have no clear designated role or mandate to enforce tobacco control policies, which could influence the little explanation of the choices that public health departments make.

The national government does not request public health departments to underpin their choices and how they plan to assess their effectiveness. Additionally, while municipalities establish smoke-free environments by placing smoke-free signs, they are not able to impose fines on individuals who violate the smoking restrictions. A similar situation occurs for smoking cessation care, as municipalities are dependent on the reimbursements and decisions of healthcare insurance providers. There are no formal rules for municipalities to negotiate with healthcare insurance providers about smoking cessation. Creating the right policy context to implement local tobacco control policies is important. For instance, in Finland, there is a nationwide data system for monitoring that is used across the country^28^. This makes enforcement of smoke-free environments easier. Additionally, Atkins et al.^32^ show that a lack of clear guidelines hinders effective communication between the national and local governments.

Second, the lack of explanation of the choices that the public health departments made could be due to limits on funding. Public health departments, reliant on municipal funding, contend with limited budgets. Therefore, the departments try to make use of every additional financial support available, aligning their proposals closely with the prescribed framework to make sure they receive the money. Each department was eligible to receive a maximum of €80000 for two years, which was equally dispersed to all municipalities. In some regions, this amounted to only €3000 per municipality, and to €13333 in others. At present, the allocated budget represents a marginal contribution in addressing the broader issue. As shown in other studies, small budgets or budget cuts may threaten the perceived importance of tobacco control at the local level^7,33^. Therefore, public health departments should either receive more funds to be able to work on three broad topics at the same time or if the budget remains small, they might be more strongly advised to allocate the funds to one topic. Since all public health departments addressed smoke-free environments, this topic could be the focus of the budget until most smoke-free child environments are smoke-free before moving on to the next topic. Alternatively, the national government could opt to reallocate budgetary resources towards cost-effective programs only with substantial health gains, similar to Denmark^6^.

Third, the lack of explanation of substantiated choices could be due to local factors such as time and knowledge. Public health departments have multiple tasks and limited time, which might have influenced how much time they could spend on this grant application. Lack of time is a common theme in the literature. For instance, Mark et al.^8^ demonstrated that insufficient time negatively impacted the implementation of smoke-free policies. Another factor could be limited knowledge. Since public health departments work on various topics, they might have limited expertise on smoking, which can affect their grant applications. These issues could also be addressed when implementing clearer guidelines at the local level.

### Strength and limitations

A strength of the study is that we included grant proposals from almost all (24 of 25) public health departments in the analysis, thereby providing a complete overview of how the public health departments in the Netherlands support local tobacco control policies. Additionally, the rational policy cycle helped us organize the data and discover the gaps in the policymaking process.

A limitation of our study is that we do not know how the activities will be executed, as this is not described in the grant applications. The grant proposals varied between 3 to 10 pages in length (1200 to 3500 words). Whether the differences between detailed and non-detailed descriptions are also real differences has not been investigated. Furthermore, as is often the case in qualitative research, the structure of the data and the type of analysis do not allow for a calculation of the intercoder reliability. The strength of employing open and axial coding lies not in achieving exact code matches across identical fragments. Instead, the power of this approach lies in recognizing discrepancies in codes and engaging in meaningful discussions surrounding them. This methodology proved instrumental in uncovering diverse interpretations of the data, facilitating discussion on these divergences, and collectively determining the most significant themes and interpretations derived from the data.

## CONCLUSIONS

The grant proposals insufficiently describe implementation strategies and process evaluation and lack adequate substantiation, nor are they evidence-informed. Under the policy conditions of the Dutch government, which included a brief explanation of the departments received, an unclear mandate, and insufficient funding, significant contributions to tobacco control policy cannot be expected from the local government. Additionally, local factors such as time and knowledge play a role. Since decentralization has been a longstanding trend, countries must carefully consider their own local policy context as well as examples from other countries when local governments work on tobacco policy. This consideration is essential for the local political arena to make a meaningful contribution to national tobacco policy.

## Data Availability

The data that support the findings of this study are available from an umbrella organization for public health departments in the Netherlands (GGD GHOR Nederland). Restrictions apply to the availability of these data: they were used under license for the current study and are not publicly available. However, data are available from the authors upon reasonable request and with permission of GGD GHOR Nederland.

## References

[1] World Health Organization. WHO Global Report on Trends in Prevalence of Tobacco Use 2000-2025 Fourth Edition. Accessed August 3, 2024.https://www.who.int/publications/i/item/9789240039322

[2] World Health Organization. WHO Report on the Global Tobacco Epidemic, 2023: Protect People from Tobacco Smoke. July 2023. Accessed August 3, 2024. https://www.who.int/publications/i/item/9789240077164

[3] Saltman RB, Bankauskaite V. Conceptualizing decentralization in European health systems: a functional perspective. Health Econ Policy Law. 2006;1:127-147. doi:10.1017/S1744133105001209 18634686

[4] Vermeulen W. Decentralisation of social policy in the Netherlands. In: Kim J, Mau NJ, eds. Decentralisation of Education, Health and Social Protection: Issues and Challenges. The Korea Institute of Public Finance and the Danish Ministry for Economic Affairs and the Interior; 2017:127-144. Accessed August 3, 2024. https://folk.ntnu.no/larseb/copenhagen_workshop_2015_pdfa.pdf

[5] Marks G, Hooghe L. Chapter Two: Contrasting Visions of Multi-Level Governance. In: Bache I, Flinders M, eds. Multi-Level Governance: Interdisciplinary Perspectives. Oxford University Press; 2003:15-30

[6] Danish Health Authority. Health Promotion Package - Tobacco. 2018. Accessed July 15, 2024. https://www.sst.dk/-/media/Udgivelser/2018/Forebyggelsespakker/103612-Forebyggelse-190x260-Tobak-UK-FINAL-WEB.ashx.

[7] Satterlund TD, Cassady D, Treiber J, Lemp C. Barriers to adopting and implementing local-level tobacco control policies. J Community Health. 2011;36(4):616-623. doi:10.1007/s10900-010-9350-6 21193951 PMC3130906

[8] Mark AJ, Sanders SC, Mitchell JA, Seale H, Richmond RL. Smoke-free outdoor areas: Supporting local government to introduce tobacco control policies. Aust N Z J Public Health. 2014;38(6):518-523. doi:10.1111/1753-6405.12265 25308696

[9] Yunarman S, Munandar A, Ahsan A, Akbarjono A, Kusuma D. Opportunities and Challenges of Tobacco Control Policy at District Level in Indonesia : A Qualitative Analysis. Asian pacafic J cancer Prev. 2021;22(10):3055-3060. doi:10.31557/APJCP.2021.22.10.3055 PMC8858251 34710979

[10] Joossens L, Olefir L, Feliu A, Fernandez E. The Tobacco Control Scale 2021 in Europe; 2022. Accessed August 3, 2024. https://www.tobaccocontrolscale.org/

[11] Nederlandse Voedsel - en Warenautoriteit. Rookverbod: Algemene informatie. Roken en Tabak. [Smoking ban: General information. Smoking and Tobacco.] Accessed July 15, 2024. https://www.nvwa.nl/onderwerpen/roken-en-tabak/rookverbod

[12] Loket Gezond Leven. Landelijk beleid tabakspreventie [National policy tobacco prevention]. Accessed July 15, 2024. https://www.loketgezondleven.nl/gezondheidsthema/roken/landelijk-beleid

[13] Ministery of Health Welfare and Sports. The National Prevention Agreement. July 2019. Accessed August 3, 2024. https://www.government.nl/documents/reports/2019/06/30/the-national-prevention-agreement.

[14] Boer M, Van Dosselaer S, De Looze M, De Roos S, Brons H, Van den Eijnden K, et al. Gezondheid en Welzijn van Jongeren in Nederland. [Health and Wellbeing of Young people in the Netherlands]. 2022. Accessed August 3, 2024. https://www.trimbos.nl/aanbod/webwinkel/af2022-hbsc-2021/

[15] Bommelé J, Hipple Walters B, Willemsen M. Smoking in the Netherlands: Key Statistics for 2022 Statistics on Smoking, Smoking Cessation, and the use of E-Cigarettes in the Netherlands. 2023. Accessed August 3, 2024. https://www.trimbos.nl/wp-content/uploads/2023/08/AF2091-Smoking-in-the-Netherlands-key-statistics-for-2022.pdf

[16] Arnold D. The Netherlands presents a plan to reduce tobacco retail outlets. Accessed August 3, 2024. https://ash.org/dutch-reduce-tobacco-retail-outlets/

[17] De Rijksoverheid. Rijksoverheid decentralisatie. Decentralisatie van overheidstaken naar gemeenten. [Central government decentralization. Decentralization of government tasks to municipalities.] Published 2022. Accessed August 3, 2024. https://www.rijksoverheid.nl/onderwerpen/gemeenten/decentralisatie-vanoverheidstaken-naar-gemeenten

[18] Mulder J, Bommelé J, Branderhorst D, Van Hasselt N. De Rookvrije Generatie Als Kans Voor Gemeenten [The Smoke-Free Generation as a Chance for Municipalities]. Accessed August 3, 2024. https://www.trimbos.nl/wp-content/uploads/sites/31/2021/09/af1591-de-rookvrije-generatie-als-kans-voor-gemeenten.pdf

[19] GGD GHOR. GGD uitleg. Wat doet een GGD. [Public health department explanation. What does a public health department do] 2022. Accessed August 3, 2024. https://ggdghor.nl/home/wat-doet-een-ggd/

[20] Huijsman F, van der Meer RM, de Beer MA, van Emst AJ, Willemsen MC. Decentralisatie van tabaksontmoediging: rookbeleid tussen wal en schip? [Decentralization of Tobacco Discouragement: Smoking Policy Falling Through the Cracks?] Tijdschr voor gezondheidswetenschappen. 2013;91(1):52-59. doi:10.1007/s12508-013-0020-9

[21] Bruijn LM, Hessels JA. Landelijke ambities versus lokale uitwerking : gemeentelijk gezondheidsbeleid en lokale tabaksontmoediging laten te wensen over. [National Ambitions versus Local Implementation: Municipal Health Policy and Local Tobacco Discouragement Leave Much to Be Desired.] Tijdschr voor gezondheidswetenschappen. 2022. doi:10.1007/s12508-022-00354-x

[22] Jansen M. Mind the Gap: Collaboration between Practice, Policy and Research in Public Health. Datawyse / Universitaire Pers Maastricht; 2007. Accessed August 3, 2024. https://www.academischewerkplaatslimburg.nl/wp-content/uploads/Proefschrift_Mind_The_Gap_Maria_Jansen.pdf

[23] Bekker MPM, Putters K, Van Der Grinten TED. Exploring the relation between evidence and decision-making: A political-administrative approach to health impact assessment. Environ Impact Assess Rev. 2004;24(2):139-149. doi:10.1016/j.eiar.2003.10.004

[24] Kuijpers TG, Kunst AE, Willemsen MC. Policies that limit youth access and exposure to tobacco: A scientific neglect of the first stages of the policy process. BMC Public Health. 2019;19(1):1-8. doi:10.1186/s12889-019-7073-x 31242893 PMC6595563

[25] GGD GHOR. Een Rookvrije Generatie in 2040. [A smoke-free generation in 2040.] Published 2019. Accessed August 3, 2024. https://www.rookvrijegeneratie.nl/gemeente/

[26] Gobo G, Molle A. Doing Ethnography. 2nd edition. SAGE; 2017

[27] Bommelé J, Hipple Walters B, Willemsen M. Local Tobacco Control Policies in the Netherlands. 2020. Accessed August 3, 2024. https://www.trimbos.nl/aanbod/webwinkel/product/af1751-localtobacco-control-policies-in-the-netherlands

[28] Mlinarić M, Hoffmann L, Lindfors P, et al. Enhancing implementation of smoke-free places: A comparative qualitative study across seven European cities. Soc Sci Med. 2020;247(October 2019):112805. doi:10.1016/j.socscimed.2020.112805 32004999

[29] European Commission. Overview of Smoke-Free Legislation and Its Implementation in the EU. 2016. Accessed August 3, 2024. https://health.ec.europa.eu/system/files/2016-11/smokefree_legislation_overview_en_0.pdf

[30] Mlinarić M, Hoffmann L, Kunst AE, et al. Explaining Mechanisms That Influence Smoke-Free Implementation at the Local Level: A Realist Review of Smoking Bans. Nicotine Tob Res. 2019;21(12):1609-1620. doi:10.1093/ntr/nty206 30285126

[31] Dutch Healthcare Authority. Informatiekaart Aanspraak en bekostiging stoppen-met-rokenzorg. Accessed August 3, 2024. https://puc.overheid.nl/nza/doc/PUC_322884_22/1/

[32] Atkins L, Kelly MP, Littleford C, Leng G, Michie S. Reversing the pipeline? Implementing public health evidence-based guidance in english local government. Implement Sci. 2017;12(1):1-13. doi:10.1186/s13012-017-0589-5 28499393 PMC5429536

[33] Anderson WJ, Cheeseman H, Butterworth G. Political priorities and public health services in English local authorities: the case of tobacco control and smoking cessation services. J Public Health (Oxf). 2018;40(3):e269-e274. doi:10.1093/pubmed/fdx143 29059319 PMC6166588

